# Emotional Reactivity in Adolescents With Non-suicidal Self-injury and Its Predictors: A Longitudinal Study

**DOI:** 10.3389/fpsyt.2022.902964

**Published:** 2022-07-08

**Authors:** Stephanie Kandsperger, Daniel Schleicher, Angelika Ecker, Florentina Keck, Sebastian Bentheimer, Romuald Brunner, Irina Jarvers

**Affiliations:** Clinic of Child and Adolescent Psychiatry, Psychosomatics and Psychotherapy, University of Regensburg, Regensburg, Germany

**Keywords:** emotional reactivity, self-injurious behavior, non-suicidal self-injury, reinforcement functions, emergency, adolescents

## Abstract

**Background:**

The management of emergency presentations at child and adolescent psychiatric outpatient clinics, by children and adolescents with self-injurious thoughts and behaviors, represents very responsible tasks but also offers the opportunity for immediate interventions. The stability and degree of emotional reactivity (ER) is a significant psychopathological symptom for development and maintenance of self-injurious behavior, differentiating between those who have continued to injure themselves and those who have not. In general, the relationship between ER and self-injurious behavior has been shown to be bidirectional. However, the stability of ER over time, as well as important predictors for ER itself have not been investigated so far. Therefore, this present study aimed at investigating the stability of ER over time and the relationship between non-suicidal self-injury (NSSI) and ER. Reinforcement functions and several variables of psychological functioning were considered as possible influencing factors.

**Methods:**

As part of a longitudinal study, 97 adolescents aged 11–18 years who presented due to self-injurious thoughts or behaviors underwent standardized emergency management. This included a specified detailed psychiatric assessment at baseline (including the Emotion Reactivity Scale, ERS, and the Self-Injurious Thoughts and Behaviors Interview, SITBI) and treatment recommendations. These were followed by a catamnestic examination with two follow-up appointments. Changes over time in ER, NSSI, reinforcement functions of NSSI and general indicators of psychological functioning (General Severity Index, GSI) were examined and significant correlations were followed up by a linear-mixed effect model predicting the ERS score over time.

**Results:**

Data analysis revealed a statistically significant decrease in ERS scores and GSI over time. However, reinforcement functions for and the symptomatology of NSSI did not change. Furthermore, no predictive relationship from ER to NSSI could be identified. A linear-mixed effect model predicting the ERS identified the GSI, automatic positive reinforcement (as a reinforcement function for NSSI) and age as the only significant predictors.

**Conclusion:**

Results demonstrate the importance of NSSI reinforcement functions for heightened emotional reactivity and emphasize their role as a point for therapeutic intervention by providing alternatives to NSSI and thereby possibly reducing emotional reactivity.

## Introduction

Emergency departments of child and adolescent psychiatries are frequently consulted by children and adolescents with self-injurious thoughts and behaviors, and often constitute children's first contact to the mental health care system (1–3). Self-injurious thoughts and behaviors range from suicidal thoughts and behaviors, including at least some intention to die, to non-suicidal thoughts and behaviors, among them non-suicidal self-injury (NSSI) (4). Recent findings from our emergency department showed a general increase in emergency presentations over the years 2014 to 2018, particularly for suicidal ideation and NSSI as reasons for presentation (3), as previous studies also observed (5, 6). NSSI is defined as self-injurious behavior, voluntary, direct injury or damage to body tissue (e.g., self-cutting, self-hitting, pinching, scratching, and biting) without a specific intentional suicidal behavior that is socially unacceptable (7–9). The high frequency of self-injurious behavior as a symptom among adolescents from the general population was shown in a study of adolescents from 11 European countries, which revealed an overall lifetime prevalence of direct self-injurious behavior (regardless of suicidal intent) of about 27.6%; 19.7% claimed occasional and 7.8% repeated direct self-injurious behavior (10). The 12-month prevalence of NSSI in a representative clinical sample of adolescent inpatients (age 13–26 years) has been reported to be as high as 60.0% (11). A systematic review of longitudinal studies of NSSI demonstrated that prevalence rates of NSSI reach their peak in middle adolescence (~15–17 years) and decline toward the end of adolescence/young adulthood (~18 years) (12).

Often NSSI is used as an inadequate coping strategy to reduce emotional distress (13). Using a functional view, Nock (4) suggested that self-injury is perpetuated by four possible reinforcement processes. These functions of NSSI are subdivided according to the automatic (intrapersonal) or social (interpersonal) reinforcement of the consequences, and according to whether they are positive or negative (4, 14). In addition to automatic (intrapersonal) negative reinforcement, i.e., to cause a removal of tension or some other negative affective feelings, there is the automatic (intrapersonal) positive reinforcement, i.e., achieve preferable thoughts or feelings. Interpersonal factors can also maintain NSSI, namely, on the one hand, social negative reinforcement, i.e., decrease or ending of a demanding social event, and on the other hand the social positive reinforcement, i.e., the appearance or increasing of a desired social event (4, 15). Most youths engaging in NSSI do so for automatic reinforcement reasons, although a substantial proportion also report social reinforcement functions (15). However, in a study from China, examining a group of high school students, it was shown that male adolescents who injured themselves were more likely to report social positive reinforcement as a reason than their female counterparts (16). Examining NSSI reinforcement functions in detail may help to shed light onto ways of diminishing NSSI itself.

Although NSSI attenuates notably in late adolescence, adolescents engaging in repeated NSSI seem to be at high risk of perpetuating dysfunctional emotion regulation strategies, even after cessation of NSSI (17). Thus, a large number of mental disorders show maladaptive emotion regulation (18). Female adolescents with NSSI showed significantly more lack of emotional clarity, difficulties demonstrating targeted behavior, difficulties in impulse control, and limited access to emotion regulation strategies than a healthy and a clinical control group with mental disorders without NSSI (19), which indicates that patients with NSSI show considerable emotion dysregulation (19, 20). Robinson et al. investigated the directionality of the relationship between emotion regulation and NSSI through a first systematic investigation and found evidence for a bidirectional relationship (21). This bidirectional relationship was argued to stem from (a) poor emotion regulation being a risk factor for NSSI a year later and (b) NSSI engagement predicting decreased emotion regulation later on (21). Robinson et al. concluded that this two-way risk relationship leads adolescents to engage in NSSI as a result of poor emotion regulation and may, critically, further weaken their emotion regulation skills (21).

As part of the emotion-response-process, the arising of emotions is usually associated with loosely coupled experience, behavior and physiological reactions: This means that one feels the emotions, behaves accordingly and shows physiological responses (22). This is the starting point for our fundamental understanding of emotional reactivity (22). According to Nock et al. (23), ER means “the extent to which an individual experiences emotions (a) in response to a wide array of stimuli (i.e., emotion sensitivity), (b) strongly or intensely (i.e., emotion intensity), and (c) for a prolonged period of time before returning to baseline level of arousal (i.e., emotion persistence).” Adolescence is an especially susceptible period for NSSI development because of heightened levels of emotional reactivity (ER) and impulsivity due to the brain's development (24). As of now, research has put less emphasis on increased ER, which likely predisposes to difficulties with emotion regulation (23). One of the reasons why ER can be so important is, because it can help explaining the development and the maintenance of behavioral problems (23).

In a previous publication a significant positive correlation between patients' ERS sensitivity score and the occurrence of NSSI within the last year was found and a correlation between the ERS and different types of reinforcement as a motivating factor for NSSI was detected (25). In order to explain the emergence and maintenance of NSSI, Hasking et al. have introduced the Cognitive-Emotional Model of NSSI. An essential consideration in this model were bidirectional relationships between ER, representations of self, representations of NSSI (i.e., functions) and NSSI-related cognitions (outcome expectancies and self-efficiacy expectancies), in respect to the dynamic relationships developing over time (26). ER is crucial as it has been shown to be one of the main factors differentiating individuals who stopped NSSI from those who engaged continuously throughout life (27). Young adults from a college population (self-injuring individuals and controls) were examined with regard to ER and self-injuring individuals showed higher scores on the Emotion Reactivity Scale (ERS) (28). The ERS total had a significant positive correlation with less appropriate coping skills (e.g., passive depressive reaction patterns and avoidance) and a negative correlation with adequate coping skills (e.g., active problem solving) (29). A study in Canada surveyed 1,125 young adults in their first year of study at a large academic institution and found an indirect effect of recent stressful experiences on NSSI engagement through ER (30). According to Nock et al. (23) examining the longitudinal course of ER and its expected relationship to psychopathology and self-injurious thoughts and behaviors is an interesting and likely fruitful area for research. Since most conducted studies were correlational and a few longitudinal studies suggest the presence of a bidirectional relationship between ER and NSSI, we focused on this bidirectional relationship in a highly burdened group of adolescent patients. Furthermore, the stability of ER as a trait appears to be crucial in sustaining or ending NSSI. This question of stability was an additional goal of the current study.

Through a brief intervention and assessments over three time points in the current study, scores in ER were expected to show sufficient variance to be able to (a) examine their stability over time and (b) examine the bidirectional relationship between ER and NSSI (26). In addition, the role of NSSI or reinforcement functions, but also general variables to assess psychological distress and functioning (31, 32), to determine the average severity of mental illness (33), and to assess psychological, social, and occupational functioning (34) were considered as influencing factors.

Thus, the rational of the present study was to (a) examine the stability of ER over a short period of time (including a small intervention) and (b) examine the bidirectional relationship between NSSI and ER. Correlations between ER and NSSI have already been studied (23, 28), but very few longitudinal studies have been conducted. Predicting the ER and NSSI is especially interesting because we were able to examine emergency presentations longitudinally with a very short-term follow-up investigation, aiding us to determine what factors can influence the ER/NSSI in the short-term. To our knowledge, emergency presentations due to NSSI have not yet been investigated with regard to the bidirectional relationship of ER and NSSI. We hypothesized that the bidirectional relationship between ER and NSSI, similar to the results of Robinson et al. (21), may also be found in the present group of emergency patients.

## Materials and Methods

### Participants and Recruitment

**Figure 1:**
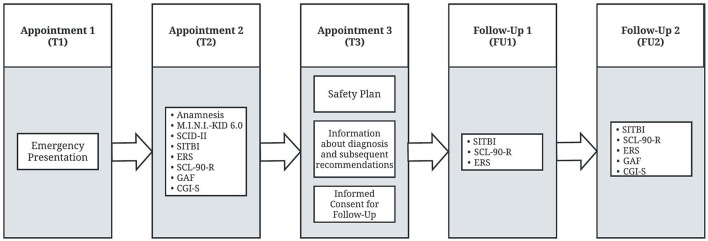
Schematic representation of the psychiatric emergency service, short-term intervention process and follow-up examinations. T1, emergency presentation due to self-injurious thoughts and behaviors; T2, standardized emergency management; T3, short-term intervention via safety plan, subsequent recommendations and informed consent for follow-up; FU1 and FU2: follow-up examinations 4 (FU1) and 8 weeks (FU2) after T3 for evaluation of the effectiveness of the standardized emergency assessment; Mini-International Neuropsychiatric Interview for Children and Adolescents (M.I.N.I. KID 6.0), Structured Clinical Interview for DSM-IV, Axis II (SCID-II), the Self-Injurious Thoughts and Behaviors Interview (SITBI), Emotion Reactivity Scale (ERS), Symptom Checklist-90 Revised (SCL-90-R), Global Assessment of Functioning Scale (GAF), Clinical Global Impressions Scale (CGI-S).

In total, 97 patients aged 11–18 years who presented as emergencies due to self-injurious thoughts and behaviors during the day and night were recruited from the emergency outpatient department of the Clinic of Child and Adolescent Psychiatry, Psychosomatics and Psychotherapy, University of Regensburg, Germany. This is a typical child and adolescent psychiatric hospital of maximum care. Data collection took place between July 2019 and April 2021. In the first emergency consultation (T1), patients received the offer of a standardized emergency management with two prompt appointments (T2 and T3), which incorporated specified diagnostic assessments (T2) and a short-term intervention (T3). The timing interval from emergency appointment, T1, to T2 was *M* = 8.15 days (*SD* = 6.11 days) and the timing interval from emergency appointment, T1, to T3 was *M* = 17.83 days (*SD* = 9.48 days). Through a specific diagnosis and early intervention in adolescents with NSSI and suicidal behavior, the purpose was to prevent a worsening of symptomatology where possible and to increase the likelihood of improvement or remission. The following group of patients was not enrolled in the study: patients with acute psychotic disorder or a different acute psychiatric status that could impair the patient's competence to agree, patients with intellectual disability according to clinical assessment, and patients who already participated in regular outpatient treatment. We also excluded patients with acute suicidal tendencies who needed prolonged inpatient treatment (more than 12 nights) in one of the hospital's inpatient units. Consequently, the sample is representative of a typical group of adolescent patients of an outpatient clinic after emergency presentation. A schematic representation of the psychiatric emergency service, short-term intervention process and follow-up examinations is shown in Figure 1.

The first emergency consultation (T1), defined standardized diagnostic measurements (T2) as well as the short-term intervention (a safety plan for handling and managing future crises was elaborated in cooperation with the adolescents) and advice on subsequent recommendations (T3) were clinical procedures. The longitudinal aspect of the study consisted of the two follow-up assessments (FU1 and FU2). At T3, patients and parents were asked whether they were willing to take part in follow-up examinations 4 and 8 weeks after T3 (FU1 and FU2) for evaluation of the effectiveness of the standardized emergency assessment. The timing interval from T3 to FU1 was *M* = 41.06 days (*SD* = 13.17 days) and from T3 to FU2 *M* = 70.98 days (*SD* = 18.65 days). If during one of the clinical presentations or the two follow-up appointments an indication for inpatient treatment was detected, it was immediately realized. Examinations for FU1 and FU2 were conducted in face-to-face contact whenever possible. However, about 40% of the examinations were conducted via telephone due to contact restrictions in the context of the COVID-19-pandemic, as well as the fact that some families lived farther away and were not able to attend face-to-face follow-up-examinations.

The presented study was approved by the Ethics Committee of the University of Regensburg (No.: 19-1426-101). Patients and their legal caregiver provided their written informed consent for study participation.

### Measures

Four clinicians from the clinic for child and adolescent psychiatry were instructed and briefed in detail for the implementation of the specific diagnostics including structured interviews and the brief intervention (T2 and T3). The two follow-up examinations were also conducted by medical doctoral students and clinical staff, who were extensively introduced to the assessment methods. The concomitant caregiver was asked about patients' date of birth/age, parents' relationship status, patients' living arrangements and patients' school type. In T2, several structured clinical interviews were implemented for dimensional and categorical psychiatric diagnostic assessment. A structured clinical interview was completed to determine diagnoses using the DSM-IV and ICD-10, namely the German Version of the Mini-International Neuropsychiatric Interview for Children and Adolescents (M.I.N.I. KID 6.0) (35). For diagnosis of a possible borderline personality disorder (BPD), also a structured clinical interview was used, namely the German version of the Structured Clinical Interview for DSM-IV, Axis II (SCID-II), subsection BPD (36). The diagnosis of BPD is affirmed if no <5 items of the 9 questions in this subsection, which are based on DSM-IV diagnostic criteria, are met (36). Two clinicians, at least including one child and adolescent psychiatric specialist, shared the results of these structured interviews applied in T2 and finally determined the final diagnoses. These were in turn communicated to the patients and accompanying caregivers in T3.

As an additional structured clinical interview, the Self-Injurious Thoughts and Behaviors Interview (SITBI) was used in T2, FU1, and FU2, which explores the presence, the severity, as well as the characteristics of self-injurious thoughts and behaviors in six subgroups (suicidal ideation, suicide plans, suicide gestures, suicide attempts, thoughts of NSSI and NSSI itself) (14). The SITBI serves as an instrument for the accurate recording of self-injurious thoughts and behaviors and can be used well in clinical and research settings, especially since the good psychometric properties of the German translation (SITBI-G) are comparable to the original interview (37). The four functions of NSSI described by Nock and Prinstein (15) are also recorded in SITBI–G (14, 37) and were queried via the following items. “Getting rid of bad feelings” measured automatic negative reinforcement (ANR); “to feel something” measured automatic positive reinforcement (APR); “to get attention” measured social positive reinforcement (SPR) and “to get out of doing something” measured social negative reinforcement (SNR) (14, 37).

A 21-item self-report for the measurement of the individual perception of ER is the Emotion Reactivity Scale (ERS), which includes 10 items for sensitivity, 7 items for arousal/intensity and 4 items for persistence on a 5-point-Likert-scale and was carried out at T2, FU1 and FU2 (23). The reliability and validity of the ERS was demonstrated by Nock et al. in a group of 87 adolescents from the community and from local psychiatric hospitals (23) and in another study in adults derived from a community screening assessment (38). In the current sample, the ERS showed a Cronbach's alpha of 0.94 for the total score, 0.88 for the sensitivity subscale, 0.89 for the intensity/arousal subscale and 0.81 for the persistence subscale, suggesting excellent internal consistency.

The German Version of the Symptom Check-List-90-R (SCL-90-R) was implemented at T2, FU1 and FU2 as an instrument to estimate psychological distress and psychological functioning (31, 32). In this self-report symptom inventory, 90 items are rated on a 5-point Likert scale from 0 to 4 (31, 32). Data about mental stress are recorded in relation to nine scales (Somatization, Obsessive-Compulsive, Interpersonal Sensitivity, Depression, Anxiety, Anger-Hostility, Phobic Anxiety, Paranoid Ideation and Psychoticism) and three global parameters (31, 32). Evidence for its validity was provided (31, 32). One of these global parameters is the Global Severity Index (GSI), which measures the average mental stress in relation to the 90 items. The GSI shows very good internal consistency (α = 0.94–0.98) (31, 32). Also, in the current sample the SCL-90 had an internal consistency of Cronbach's alpha = 0.97.

At T2 and FU2, the Clinical Global Impressions Scale—Severity (CGI-S) was rated by the experienced clinicians. This was used to estimate the average severity of the mental illness over the last 7 days based on all available information (33). The CGI-S is scored on a 7-point scale, from 0 = normal to 6 = among the most extremely ill patients (33).

Finally, to assess the general level of functioning, the experienced clinicians used the Global Assessment of Functioning Scale (GAF). This measurement at T2 and FU2 evaluated psychological, social and professional functioning. The scale is divided into 10 levels of functioning, each with 10 subpoints, and ranges in rating from 100 (highest level of performance) to 0 (lowest level of performance) (34).

### Statistical Analyses

In a first step, the relevance of control variables was examined. The effect of the control variable sex on main variables was examined via independent sample *t*-tests or Mann-Whitney *U*-tests depending on the distribution. Main variables were: patients' ERS scores, age, frequency of NSSI behavior in the past month, frequency of NSSI thoughts in the past month, intensity of NSSI thoughts in the past month and NSSI reinforcement functions. Additionally, several variables of psychological functioning were included: the GSI, GAF and CGI-S. In a second step, changes in ERS scores, NSSI reinforcement functions, frequency of NSSI behaviors, frequency of NSSI thoughts, intensity of NSSI thoughts and finally the GSI, GAF and CGI-S score were examined over the three time points using the Friedmann test, followed by *post-hoc* tests using the Sign test. Next, the relationship between main variables was analyzed through bivariate correlations (Kendall's τ). Significant correlations between the ERS and NSSI behavior at any of the time points and other main variables were used in order to identify potential predictors for the ERS score and NSSI behavior.

In a subsequent step, a linear mixed effect model (LME) was computed in order to assess which factors significantly contribute to the ERS score. As no significant correlational relationship between earlier ERS scores and subsequent NSSI behavior was identified, no such model for NSSI behavior was computed. Instead of examining separate relationships between the different time points, an LME allows for a model over all the longitudinal data while considering subject-specific differences across time as a random effect. An additional advantage of LMEs is their robustness toward missing data (39). As correlational patterns did not differ for the ERS scales, only the total score was used as an outcome variable. The general modeling strategy for the ERS model was the following:


$$\begin{array}{l} ERS_{Total}\sim Age+NSSI_{Thoughts}+Intensity_{NSSIThoughts}\\ +NSSI_{Behavior}+R_{AN}+R_{AP}+R_{SP}+R_{SN}+GSI+Time\\ +\left(Time|Subject\right) \end{array}$$

Where *ERS*_*Total*_ refers to the ERS total score, *NSSI*_*Thoughts*_ refers to the frequency of NSSI thoughts in the past month, *Intensity*_*NSSIThoughts*_ refers to the intensity of NSSI thoughts at the worst moment, *NSSI*_*Behavior*_ refers to the frequency of NSSI behavior in the past month, *R*_*AN*_ refers to automatic negative reinforcement, *R*_*AP*_ refers to automatic positive reinforcement, *R*_*SP*_ refers to social positive reinforcement, *R*_*SN*_ refers to social negative reinforcement, *Time* refers to the three time points and *(Time|Subject)* refers to the random effect of subject over time.

The model followed best practice recommendations for model-fitting (40) by computing a null model with a random slope, followed by a maximized model including all predictors. As a final step, a reduced model with significant predictors was computed and compared to the other models using χ^2^. Model fit was assessed via the Akaike Information Criterion (AIC) (41), the Bayesian Information Criterion (BIC) (42) and the log likelihood ratio (LR) statistics. The model with the lowest BIC and AIC while being statistically different from the null model and parsimonious was chosen.

The R statistical package, version 4.0.2 (43) and the *lme4* package were used in order to compute LMEs (44). The Satterthwaite approximation to degrees of freedom via the *lmerTEST* package was used in order to compute *p*-values (45) and finally, the *r2glmm* package (46) was used to provide *R*^2^ values as a measure of effect size. All other analyses were conducted using SPSS 28 (IBM Corp. Released 2021. IBM SPSS Statistics for Windows, Version 28.0. Armonk, NY:IBM Corp.). Where appropriate, the false discovery rate (FDR) was used to correct for multiple comparisons and reported *p*-values correspond to the correction (47). The statistical significance level was set to α = 0.05.

## Results

### Sample Characteristics

Detailed sociodemographic and clinical characteristics can be found in Table 1. A total of 97 children and adolescents between the ages of 11 and 18 participated in T2. The sample reduced to *n* = 68 for FU2. Additional *n* = 23 participants were part of the survey, but could not be included in the final analysis due to language difficulties (*n* = 2), terminating their participation (*n* = 4) or not exhibiting NSSI thoughts or NSSI behavior (*n* = 17). The presented data were acquired in the period from July 2019 to April 2021. Mean age at T2 was *M* = 14; 11 (years; months) (*SD* = 1; 6, range = 11; 0–18; 0) and 77.6% were female, 21.6% were male and 1% was diverse.

**Table 1:** Sociodemographic and clinical characteristics of participants.

			**Range**	**Total *N***
**Gender**	***N***	**%**		97
Female	75	77.3		
Male	21	21.6		
Divers	1	1.0		
Age	***M*** 14.90	***SD*** 1.52	11–18	97
**School type**	***N***	**%**		97
Gymnasium	25	25.8		
Realschule	25	25.8		
Mittelschule	22	22.7		
Förderschule	2	2.0		
Fach- und berufsschule	12	12.4		
Other school type/no school	6	6.2		
Unknown	5	5.2		
**Household composition**	***N***	**%**		97
With mother and/or father	79	81.4		
At institutional care	4	4.1		
Lives with partner	1	1.0		
Unknown	13	13.4		
**NSSI thoughts**	***N***	**%**		
Lifetime prevalence (T2)	93	95.9		97
Lifetime prevalence (FU1)	69	93.2		74
Lifetime prevalence (FU2)	63	92.6		68
	***M***	***SD***		
Number of episodes (lifetime) (T2)	70.00	197.01	0–1,000	92
Number of episodes (lifetime) (FU1)	75.77	169.34	1–1,000	69
Number of episodes (lifetime) (FU2)	81.68	186.93	1–1,100	63
Number of episodes (last month) (T2)	4.57	8.12	0–40	91
Number of episodes (last month) (FU1)	4.97	8.58	0–30	69
Number of episodes (last month) (FU2)	3.33	5.78	0–30	63
**NSSI intensity**	***M***	***SD***		
Intensity (worst point in time) (T2)	3.47	0.86	0–4	92
Intensity (worst point in time) (FU1)	3.43	0.78	1–4	69
Intensity (worst point in time) (FU2)	3.40	0.81	1–4	63
**NSSI behavior**	***N***	**%**		
Lifetime prevalence (T2)	88	91.7		96
Lifetime prevalence (FU1)	67	90.5		74
Lifetime prevalence (FU2)	62	91.2		68
	***M***	***SD***		
Frequency (lifetime) (T2)	116.01	245.11	0–1,000	91
Frequency (lifetime) (FU1)	86.94	161.75	0–1,000	68
Frequency (lifetime) (FU2)	66.16	107.62	1–585	62
Frequency (last month) (T2)	6.20	8.50	0–40	86
Frequency (last month) (FU1)	5.90	9.53	0–40	68
Frequency (last month) (FU2)	3.94	9.49	0–60	62

*Survey time points: baseline examination (T2), follow-up 1 (FU1), follow-up 2 (FU2); Non-suicidal self-injury (NSSI). Gymnasium (higher level education, usually 8–9 years of school after 4 years of elementary school, terminating with the general university entrance qualification), Realschule (intermediate secondary school, 6 years of school after 4 years of elementary school), Mittelschule (9 years of elementary school), Fach-/Berufsschule (2–3 years vocational training school most commonly after Mittelschule or Realschule, but also possible after Gymnasium) and Förderschule (school for special needs)*.

Girls and boys did not differ in age (*U* = 777.00, *z* = −0.09, *p* = 0.924), the ERS score at T2 (*t* = 1.08, *p* = 0.095), NSSI behavior at T2 (*U* = 504.00, *z* = −0.82, *p* = 0.409), NSSI thoughts at T2 (*U* = 670.00, *z* = −0.14, *p* = 0.885) or NSSI reinforcement functions (*U* > 460.00, *z* < −1.79, *p* > 0.05). However, they differed in their intensity of NSSI thoughts at T2 with girls showing a higher intensity (*M* = 3.60, *SD* = 0.66) than boys (*M* = 2.95, *SD* = 1.26); *U* = 491.50, *z* = −2.22, *p* = 0.026. This difference was not present for the subsequent time points FU1 (*U* = 338.00, *z* = −1.10, *p* = 0.270) and FU2 (*U* = 315.50, *z* = −0.53, *p* = 0.596).

The ICD-10 distribution of psychiatric diagnoses (listed in order of frequency) is as follows: F3 [Mood (affective) disorders], *n* = 85 (82.45%); F4 (Neurotic, stress-related and somatoform disorders), *n* = 56 (54.32%); F9 (Behavioral and emotional disorders with onset usually occurring in childhood and adolescence), *n* = 31 (30.07%); F1 (Psychological and behavioral disorders caused by psychotropic substances), *n* = 10 (9.70%); F6 (Disorders of adult personality and behavior), *n* = 6 (5.82%); F5 (Behavioral syndromes associated with physiological disturbances and physical factors), *n* = 4 (3.88%) and F8 (Disorders of psychological development), *n* = 3 (2.91%). There were multiple diagnoses possible per patient. Two patients fulfilled the diagnostic criteria of BPD according to DSM-IV.

### Changes Over Time

**Figure 2:**
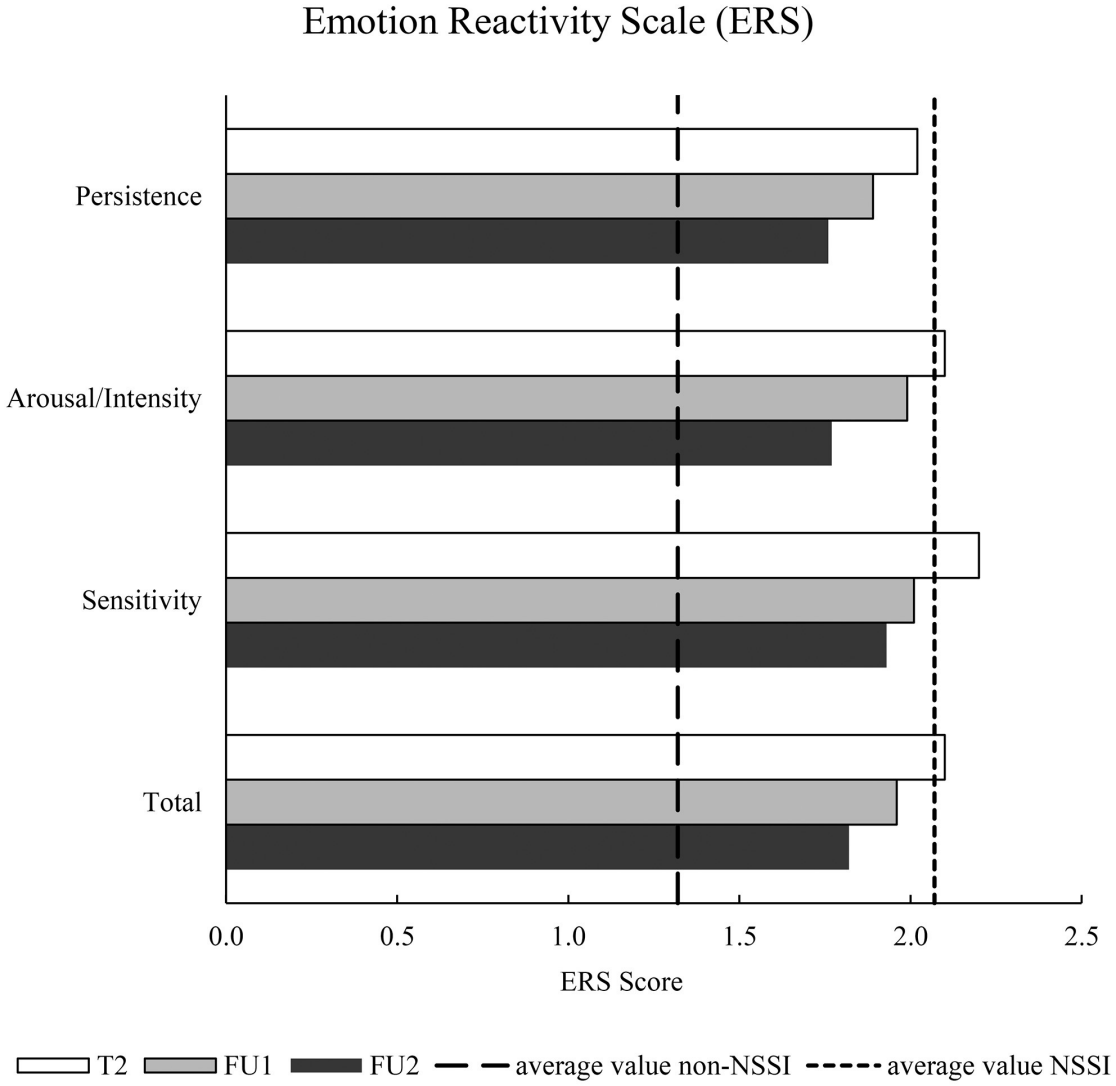
Descriptive Emotion Reactivity Scale results over all examination time points. Survey time points: baseline examination (T2), follow-up 1 (FU1), follow-up 2 (FU2); Non-suicidal Self-injury (NSSI); average values for (non) NSSI ERS scores correspond to group means from Nock et al. (23).

A statistically significant difference in ERS scores over the three time points could be observed, $\chi_{\left(2\right)}^{2}$ = 8.95, *p* = 0.011, *n* = 68. FDR-corrected *post-hoc* tests revealed a significant difference between ERS scores at T2 and FU2 (*z* = −2.30, *p* = 0.049, *n* = 68), but not between T2 and FU1 (*z* = −2.14, *p* = 0.063, *n* = 73) and FU1 and FU2 (*z* = −0.85, *p* = 0.396, *n* = 68). Examining the three ERS scales independently, revealed significant change for the sensitivity scale [$\chi_{\left(2\right)}^{2}$ = 10.98, *p* = 0.004, *n* = 68], the intensity/arousal scale [$\chi_{\left(2\right)}^{2}$ = 6.28, *p* = 0.043, *n* = 68] but not for the persistence scale [$\chi_{\left(2\right)}^{2}$ = 1.96, *p* = 0.375, *n* = 68]. Overall, a decrease could be observed over time (see Figure 2).

**Figure 3:**
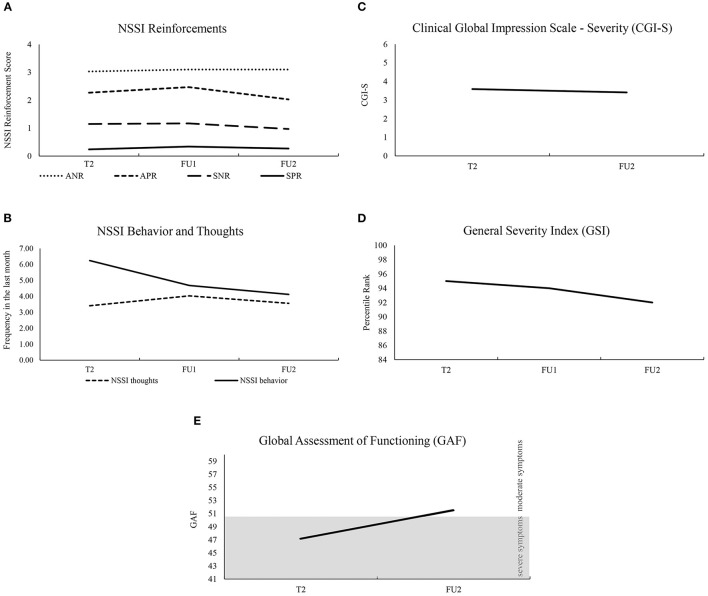
Descriptive presentation showing results over all examination time points. Survey time points: baseline examination (T2), follow-up 1 (FU1), follow-up 2 (FU2); Non-suicidal Self-injury (NSSI); NSSI Reinforcements: Automatic Negative Reinforcement (ANR), Automatic Positive Reinforcement (APR), Social Negative Reinforcement (SNR), Social Positive Reinforcement (SPR); scale range for NSSI reinforcements was between 0 and 4 **(A)**, scale range for NSSI thoughts and behavior **(B)**, scale range for CGI-S was between 0 and 6 **(C)**, was chosen from mean values, maximum values were (0; 30) for NSSI thoughts and (0; 60) for NSSI behavior; scale range for GSI **(D)** was chosen from the range of clinical abnormality (percentile rank corresponds to T score >60) to the maximum; scale range for GAF **(E)** represents the two clinically relevant severity levels “severe symptoms” (41; 50) and “moderate symptoms” (51; 60).

For the deployment of NSSI reinforcement functions, there was no statistically significant difference for either of the reinforcement functions; automatic negative reinforcement [$\chi_{\left(2\right)}^{2}$ = 1.67, *p* = 0.434, *n* = 59], automatic positive reinforcement [$\chi_{\left(2\right)}^{2}$ = 4.56, *p* = 0.102, *n* = 59], social positive reinforcement [$\chi_{\left(2\right)}^{2}$ = 2.82, *p* = 0.244, *n* = 59] or social negative reinforcement [$\chi_{\left(2\right)}^{2}$ = 0.99, *p* = 0.609, *n* = 59]. See Figure 3A for a descriptive overview.

Also, for the frequency and intensity of NSSI thoughts and NSSI behavior in the last month, there was no significant difference over T2, FU1 and FU2: NSSI thoughts: $\chi_{\left(2\right)}^{2}$ = 0.36, *p* = 0.836, *n* = 59; intensity of NSSI thoughts: $\chi_{\left(2\right)}^{2}$ = 0.30, *p* = 0.860, *n* = 58; NSSI behavior: $\chi_{\left(2\right)}^{2}$ = 5.01, *p* = 0.078, *n* = 59 (see Figure 3B).

The GSI showed differences over time, $\chi_{\left(2\right)}^{2}$ = 10.98, *p* = 0.004, *n* = 65 and *post-hoc* tests revealed a significant difference between the GSI between T2 and FU2 (*z* = −2.86, *p* = 0.004, *n* = 68), but not between T2 and FU1 (*z* = −1.26, *p* = 0.208, *n* = 72) or FU1 and FU2 (*z* = −1.18, *p* = 0.237, *n* = 68). The GAF and the CGI-S were only administered at T2 and FU2. Both showed a difference over time with an increase in the GAF (*z* = −4.87, *p* < 0.001, *n* = 68) and a decrease in the CGI (*z* = −2.50, *p* = 0.013, *n* = 68). See Figures 3C–E for a descriptive overview.

### Correlations Between Main Variables

All correlations between the ERS at FU1 and FU2 and main variables across all three time points are depicted in Table 2. Variables significantly correlated with the ERS total score at FU1 and/or FU2 were age, NSSI thoughts (at T2, FU1 and FU2), intensity of NSSI thoughts (at T2, FU1 and FU2), NSSI behavior (at FU1 and FU2), automatic positive reinforcement (at FU1 and FU2), social negative reinforcement (at T2), the CGI-S (at FU2), GAF (at T2 and FU2) and the GSI (at T2, FU1 and FU2). These variables were considered possible predictors of the ERS. As the CGI-S, GAF and GSI were highly correlated, only the GSI (as the factor with the strongest correlation) was entered as a predictor. Correlational relationships among predictor variables were present, but never exceeded an r of 0.50.

**Table 2:** Overview of Kendall's τ correlations between the ERS at FU1 and FU2 and other main variables across the time points T2, FU1 and FU2.

	**NSSI thoughts** **T2**	**Intensity NSSI thoughts** **T2**	**NSSI behavior** **T2**	**Automatic Negative reinforcement** **T2**	**Automatic positive reinforcement** **T2**	**Positive social reinforcement** **T2**	**Negative social reinforcement** **T2**	**CGI-S** **T2**	**GAF** **T2**	**GSI-S** **T2**	
ERS FU1	0.223*	0.160	0.020	0.030	0.013	0.141	0.241*	0.133	−0.145	0.393**	τ
	68	68	64	65	65	64	64	73	73	72	*N*
ERS FU2	0.230*	0.271**	0.143	0.046	0.114	0.098	0.202	0.203	−0.216*	0.437**	τ
	63	63	59	60	60	59	59	68	68	67	*N*
	NSSI thoughts FU1	Intensity NSSI thoughts FU1	NSSI behavior FU1	Automatic negative reinforcement FU1	Automatic positive automatic reinforcement FU1	Automatic positive social reinforcement FU1	Automatic negative social reinforcement FU1	GSI-S FU1			
ERS FU1	0.254**	0.313**	0.131	0.094	0.137	0.090	0.117	0.463**	τ		
	68	68	67	66	66	66	66	71	*N*		
ERS FU2	0.290**	0.417**	0.266**	0.134	0.216*	0.130	0.190	0.483**	τ		
	63	63	62	61	61	61	61	66	*N*		
	NSSI thoughts FU2	Intensity NSSI thoughts FU2	NSSI behavior FU2	Automatic negative reinforcement FU2	Automatic positive reinforcement FU2	Automatic positive social reinforcement FU2	Automatic negative social reinforcement FU2	CGI-S FU2	GAF FU2	GSI-S FU2	
ERS FU1	0.324**	0.237	0.142	0.189	0.200	0.074	0.116	0.226	−0.211*	0.385**	τ
	63	63	62	62	62	62	62	68	68	68	*N*
ERS FU2	0.429**	0.287**	0.332**	0.185	0.287**	0.027	0.191	0.365**	−0.327**	0.553**	τ
	63	63	62	62	62	62	62	68	68	68	*N*

Survey time points: baseline examination (T2), follow-up 1 (FU1), follow-up 2 (FU2); ERS, emotion reactivity scale; NSSI, Non-suicidal self-injury; CGI-S, clinical global impression scale—severity; GAF, global assessment of functioning; GSI, general severity index. Significance after FDR correction is reported. *p < 0.05, **p < 0.01.

To examine which variables may be predictors of NSSI behavior at FU1 and/or FU2, correlations with main variables across T2 and F1 were examined. NSSI behavior correlated significantly with the frequency of NSSI thoughts (F1: *r* = 0.48 *p*_FDR_ = 0.002), the intensity of NSSI thoughts (T2: *r* = 0.29, *p*_FDR_ = 0.015; FU1: *r* = 0.221, *p*_FDR_ = 0.048), the GSI (T2: *r* = 0.22, *p*_FDR_ = 0.021; FU1: *r* = 0.32, *p*_FDR_ = 0.003) and automatic positive reinforcement (F1: *r* = 0.29, *p*_FDR_ = 0.028). There was no significant correlation with the ERS or any of the subscales after correcting for multiple comparisons (*r* = 0.08–0.20, *p*_FDR_ > 0.05). As NSSI behavior showed little variance over time and no significant correlations with the ERS could be identified, no model predicting NSSI behavior was computed.

### Linear Mixed Effect Model Prediction the ERS

In a first step, a null model was computed with a random intercept of subject, followed by a null model with a random slope of time over subject. The null model with a random slope performed better than the intercept model, therefore it was chosen as the comparison model.

A linear mixed effect model was computed with the ERS total score as the outcome variable and age, NSSI thoughts, intensity of NSSI thoughts, NSSI behavior, the four types of reinforcement, the GSI and time as possible predictors. Additionally, a random slope of subject over time was added as a random effect. The model performed significantly better than the null model (see Table 3). Significant predictors of the ERS total score over time were the GSI, automatic positive reinforcement and age. These predictors were entered into a reduced model which showed the same pattern and had lower model fit indices than the maximized model. See Table 4 for an overview of the fixed effects of the reduced model.

**Table 3:** Model comparisons of linear-mixed effect models predicting the ERS total score.

**Outcome variable**	**Model**	**AIC**	**BIC**	**logLik**	**Vs. null model**		**Vs. max model**	
					***X* ^2^**	***P*-value**	***X* ^2^**	***P*-value**
ERS total score	Null model (Intercept)	873.32	886.55	−432.66				
	Null model (Slope)	866.84	886.69	−427.42	10.48	0.005		
	Max model	775.37	824.99	−372.69	109.47	<0.001		
	Reduced model	768.90	795.36	−376.45	112.42	<0.001	7.53	0.376

*AIC, akaike information criterion; BIC, bayesian information criterion; logLik, log-likelihood; ERS, emotion reactivity scale*.

**Table 4:** Linear mixed effect model predicting the ERS with fixed effects.

**Outcome variable**	**Fixed effect**	**Estimate**	***SD***	***T*-value**	***P*-value**	$R_{\beta *}^{2}\;$	$R_{\beta *}^{2}\;$ Cl
ERS total score	Age	0.39	0.12	3.34	0.001	0.07	0.02–0.16
	Automatic positive reinforcement	0.25	0.08	3.03	0.003	0.01	0.00–0.06
	GSI	2.28	0.20	11.33	<0.001	0.33	0.24–0.43

For individual model terms, the semi-partial (marginal) R^2^ ($R_{\beta *}^{2}\;$) is reported including confidence intervals. The reduced model is depicted. ERS, emotion reactivity scale; GSI, global severity index.

## Discussion

As part of a longitudinal study, the present study (a) examined the stability of emotional reactivity and (b) examined the bidirectional relationship between NSSI and emotional reactivity over a short period of time (including a small intervention). In particular, we were able to take a closer look at the time course of ER, (intensity of) NSSI thoughts, and NSSI behavior, as well as the underlying reinforcement functions for NSSI in adolescents who presented as emergency outpatients to a child and adolescent psychiatric clinic.

Over time, there was a significant decrease in ERS from T2 to FU2 (not to FU1); among the subscales of ERS, this pattern was evident with regard to the sensitivity and intensity/arousal scales, while persistence did not show a significant reduction over time. In our group of adolescent patients, persistence, i.e., ER that persists for a prolonged period of time until returning to baseline levels of arousal, remained unchanged. In contrast, sensitivity, i.e., ER with respect to a wide range of stimuli, and intensity/arousal, i.e., the strength or intensity of the emotional response, decreased significantly. For this reason, one could assume that the persistence of ER is the most stable and thus, slowest and, perhaps, most resistant with regard to change. Furthermore, the three parameters (1) GSI as an indicator of general mental distress (31, 32), (2) GAF as a general level of functioning (34), and (3) CGI-S as an indicator of the severity of the patient's illness (33) changed between T2 and FU2 to the benefit of the patients. However, this change over time was not observed in the frequency of NSSI thoughts, NSSI behavior, and in the intensity of NSSI thoughts; nor did the NSSI reinforcement functions change significantly. Overall, improvement was observed in general psychopathology and emotional reactivity, whereas no improvement in the specific NSSI symptomatology took place. This demonstrates that other behavioral, environmental, emotional, cognitive, and biological factors are certainly involved (23). Overall, improvements are unlikely to occur over such a short period of time as there is evidence of an increase in NSSI rates in adolescence, especially early adolescence with a decrease in young adulthood (12). Thus, previous studies have already distinguished between episodic and repeated or chronic NSSI (48, 49). In addition, the follow-up period in our study was relatively short, so that, also according to our clinical experience, it usually takes a longer time for adolescents to be able to stop self-injuring. However, a missing effect may also have been due to insufficient power as frequencies decreased descriptively. Finally, the fact that NSSI reinforcement functions did not change over time suggests that they are stable and not characterized by rapid change.

Apart from changes over time, possible predictors of NSSI and ER were examined. No bidirectional relationship between ER and NSSI could be identified. In particular, ER was not predictive of NSSI, whereas NSSI behavior was among the variables significantly correlating with ER. The specific directionality identified in our sample may be due to several factors: On the one hand, the period examined was rather short, thus, little variance in NSSI behavior could be observed over time. On the other hand, Dawkins et al. (50) have shown that the relationship between ER and NSSI is present particularly when patients had low self-efficacy in refraining from NSSI. The present sample consisted of emergency presentations, which suggests that patients must have assumed or at least hoped to receive help and thereby improve their NSSI symptomatology. Thus, a certain conviction that stopping NSSI behavior is possible may have been present. However, as NSSI appeared to be a possible predictor for ER, the opposite direction could be examined in more detail. Age, automatic positive reinforcement and the GSI were identified as significant predictors of ER over time.

Nock et al. (23) developed and tested the ERS and observed that scores on the ERS were not significantly associated with age. In this previous study, adolescents and young adults were recruited from the community and from the local psychiatric hospital with a mean age of 17; 2 (years; months) (23). The mean age in our study is lower [14; 11 (years; months)] and participants are a group of adolescents from the emergency department focusing on self-injurious thoughts and behaviors. The younger age and the examination of clearly burdened patients could explain why, in our case, increasing age predicts a higher ER. Older patients may have also been suffering from NSSI longer than younger patients.

Despite most adolescents naming automatic negative reinforcement as the most common function for NSSI (15, 51), automatic positive reinforcement should not be discarded. Automatic positive reinforcement has been less the focus of previous research, but is also frequently reported as a NSSI function (52). In our longitudinal study, the NSSI reinforcement function of automatic positive reinforcement predicts ER, i.e., those adolescents who report more automatic positive reinforcement as a reason for NSSI showed higher ER. Through automatic positive reinforcement, NSSI is utilized to provoke sensations or feelings that enhance and reinforce this symptomatology as a result (15, 51). This could be interpreted in such a way that adolescents who cannot cope with a feeling of numbness or emptiness and therefore hurt themselves, also show a higher ER and this higher ER can also be predicted for later time points. This in turn could mean that they provoke or push themselves emotionally through NSSI, struggle to cope with it accordingly and as a result show an increased ER. In a study from the US, 30 adolescents with NSSI were explored and divided into groups with automatic positive reinforcement and without this reinforcement function. Individuals with NSSI due to automatic positive reinforcement reported that they had more NSSI thoughts, longer duration of these thoughts, and more NSSI behaviors (52). More than 50% of the sample reported engaging in at least one case of NSSI for this reinforcement function (52). In a study with adolescents admitted to an inpatient psychiatric department for suicidal behaviors, adolescents who stated automatic positive reinforcement as the main reason for NSSI demonstrated a higher likelihood of continuing to commit NSSI over 6 months (53). The third factor that significantly predicted ER over time, and this very clearly, was the GSI (Global Severity Index) from the SCL-90 as a general indicator of mental distress (31, 32). The GSI measures general psychological stress in the past 7 days; thus, the GSI can be seen as the best indicator of the current extent of overall mental stress present, because it relates the intensity of stress to all 90 items (32). This indicates that general mental stress resulting from a variety of areas may subsequently increase ER. This is a more general relationship and difficult to address therapeutically, however, as increased ER makes continuous NSSI more likely (27), reducing general mental stress may already be an aid in reducing the need for NSSI. Patients may feel mentally burdened which leads to heightened emotional reactivity, which is in turn expressed through NSSI (for as long as NSSI is considered a valid problem-solving strategy).

Although this is a longitudinal study with patients presenting during an acute crisis, some limitations should be mentioned. The assessment of the patients' symptomatology was mainly based on the patients' self-assessment (e.g., SITBI, ERS, SCL-90) and may suffer from bias. However, the self-assessments chosen showed high internal consistency and have been validated previously (23, 31, 32, 37, 38). Two follow-up examinations were performed, which, however, were conducted at a rather short distance from each other (4 weeks and 8 weeks after appointment 3). For a further investigation, it would certainly be important to investigate the bidirectional relationship between NSSI and ER in a highly burdened sample with more variance in NSSI over a longer period of time than in our study. The short follow-up period was chosen because, in addition to the organizational challenges, the follow-up appointments were intended to serve as a reminder of the recommendations made and to improve compliance. Furthermore, we intended to use the initial motivation to attend further appointments (as promptly as possible), because in our clinical experience, clients presenting during an acute crisis are often no longer willing to attend appointments in the clinic over a longer period of time. For NSSI symptomatology, a longer follow-up interval could be useful in a future study in order to give time for patients to improve. Another limitation may be that the current sample does not represent the most severely burdened patients. Since this was an outpatient emergency management, patients with longer inpatient treatment or already existing outpatient care were excluded. Also inpatients who had been in treatment for a longer period of time were excluded. It could be assumed that the involvement of this subgroup would probably have led to a more severe psychopathology and psychological burden. Nevertheless, this is a utilization population of outpatients with self-injurious thoughts and behaviors who have not or not sufficient outpatient care received so far. Finally, due to contact restrictions in the context of the COVID-19-pandemic, as well as the fact that some families lived farther away and were not able to attend to face-to-face follow-up-examinations, several appointments had to take place over the phone. Some patients may have hesitated more and responded less truthfully in these interviews; opposed to filling out questionnaires by themselves.

A major strength of our study is the respectable sample of emergency patients, who were comprehensively characterized by a standardized diagnostic procedure and followed up by two follow-up examinations. The examinations were carried out by well-trained clinicians and medical doctoral students, the consultation and determination of psychiatric diagnoses as well as recommendations were carried out by a child and adolescent psychiatric specialist in consultation with the aforementioned colleagues. Adolescents with self-injurious thoughts and behaviors can be difficult to motivate to participate in outpatient care and should be provided with treatment opportunities when they are presented as emergencies due to self-injurious thoughts and behaviors. Through this present standardized outpatient emergency management program, adolescents with self-injurious thoughts and behaviors were able to start their treatment procedure as quickly as possible and thereby reduce the likelihood of chronification of symptoms and further emergency presentations.

In this present study, adolescents with NSSI were examined over a longer period of time and factors influencing ER were identified, which has not been investigated up to now. In the future, the risk of automatic positive reinforcement, a high GSI and age can be considered for the development of ER. Especially as ER plays a crucial role in stopping or sustaining NSSI, it may provide an option for early intervention and further research initiatives. From a clinical perspective, influencing ER itself appears difficult, especially with young children. Instead, reducing behavior while in a clinical context as well as teaching alternative methods to achieve goals that have been previously achieved via NSSI (presenting self-efficacy through other means) appears to be a more promising starting point. This starting point may subsequently aid reducing ER—a crucial factor differentiating between patients who continuously engage in NSSI and those who stop (27).

## Data Availability Statement

The raw data supporting the conclusions of this article will be made available by the authors, without undue reservation.

## Ethics Statement

The studies involving human participants were reviewed and approved by Institutional Examination Board for the Medical Faculty of the University of Regensburg. Written informed consent to participate in this study was provided by the participants' legal guardian/next of kin.

## Author Contributions

SK and RB initiated the idea for this study and designed the study. IJ provided contributions to the hypotheses, sample size calculation, and statistical analyses. The first manuscript was drafted by SK and IJ. DS and AE were engaged in the planning and coordination of the study. FK and SB conducted data collection with patients at FU1 and FU2. All authors read and approved the final manuscript.

## Funding

This research is funded by the Medical Faculty at the University of Regensburg and the Clinic of Child and Adolescent Psychiatry, Psychosomatics and Psychotherapy under the direction of RB. The study design, the collection, analysis, and interpretation of the data as well as the preparation of the manuscript are not financed externally.

## Conflict of Interest

The authors declare that the research was conducted in the absence of any commercial or financial relationships that could be construed as a potential conflict of interest.

## Publisher's Note

All claims expressed in this article are solely those of the authors and do not necessarily represent those of their affiliated organizations, or those of the publisher, the editors and the reviewers. Any product that may be evaluated in this article, or claim that may be made by its manufacturer, is not guaranteed or endorsed by the publisher.
